# Pathological severity determines the renal recovery for anti-myeloperoxidase antibody-associated vasculitis requiring dialysis at disease onset: a retrospective study

**DOI:** 10.1186/s12882-019-1487-7

**Published:** 2019-07-30

**Authors:** Peng-cheng Xu, Tong Chen, Si-jing Wu, Xia Yang, Shan Gao, Shui-yi Hu, Li Wei, Tie-kun Yan

**Affiliations:** 1 Department of Nephrology, Tianjin Medical University General Hospital, Tianjin, 300052 China; 2 Department of Hematology, Tianjin Medical University General Hospital, Tianjin, 300052 China

**Keywords:** Antineutrophil cytoplasmic antibody, Myeloperoxidase, Renal biopsy, Histopathologic classification, Dialysis

## Abstract

**Background:**

Many patients with anti-neutrophil cytoplasmic antibody (ANCA)-associated vasculitis (AAV) need dialysis at disease onset due to severe kidney injury. Determining whether they can become dialysis independent is an important clinical assessment.

**Methods:**

Forty kidney biopsy-proved myeloperoxidase (MPO)-ANCA associated AAV patients who required dialysis at disease onset were enrolled. Relationships between laboratory and pathological characteristics and prognoses were analyzed.

**Results:**

Twenty-five patients obtained dialysis independence within 3 months, while the other 15 patients remained dialysis dependent. No sclerotic class was identified among the 40 patients. Only two biopsies exhibited focal class diagnoses and both these patients recovered their renal function. The renal recovery rate of the 20 patients with mixed class was significantly lower than that of the 18 patients with crescentic class (40.0% vs. 83.3%, *p* = 0.006). Receiver operating characteristics (ROC) curves showed fibrous crescent+global glomerulosclerosis greater than 32.6% was a strong predictor of dialysis dependence with a sensitivity of 93.3% and specificity of 88.0%. When the percentage of fibrous crescent+global glomerulosclerosis exceeded 47.9%, dialysis independence was not possible. Correlation analysis indicated that platelet counts were negatively correlated with the percentage of fibrous crescent+global glomerulosclerosis (R = -0.448, *p* = 0.004). Most patients with increased platelets (84.62%) obtained renal recovery. Compared with methylprednisolone pulse therapy, plasma exchange accelerated renal recovery (29.4 ± 15.6 vs. 41.4 ± 11.7 days, *p* = 0.039).

**Conclusions:**

For MPO-ANCA AAV who required dialysis at disease onset, crescentic and mixed classes accounted for the majority of patients in our cohort. The renal outcome of mixed class patients was worse than that of crescentic class. A high proportion of fibrous crescent+global glomerulosclerosis is a predictor of dialysis dependence. Increased platelet count is associated with active and reversible renal lesions.

## Background

Anti-neutrophil cytoplasmic antibody (ANCA)-associated vasculitis (AAV) is a systemic vasculitis. The kidney injury manifests as a rapidly progressive glomerulonephritis (RPGN), and this is very common in AAV and affects disease prognosis [1, 2]. Kidney biopsy typically reveals pauci-immune necrotizing glomerulonephritis (GN) [3, 4]. In AAV, a percentage of patients require renal replacement therapy at disease onset. Some of these patients enter end stage renal disease (ESRD) rapidly in spite of intensive immunosuppressive therapy [5].

Without treatment, the prognosis of AAV is very poor. Immunosuppressive therapy effectively increases survival. However, since intensive immunosuppressive therapy can lead to fatal infection [6], reducing the intensity of immunosuppressive therapy is necessary for patients who will not recover renal function. Based on the results of previous studies, plasma exchange (PE) could increase the rate of renal function recovery in AAV [7], but not all patients recover renal function with PE. Determining whether patients with severe kidney injury have the potential to become dialysis independent is of great importance in clinical practice.

According to the pathological classification proposed by Berden AE et al. [8], the pathological manifestations of ANCA-GN can be classified as focal, crescentic, mixed and sclerotic categories. This classification has been demonstrated to be effective in predicting the risk of ESRD, but there are no studies on whether the pathological characteristics can be used to assess the possibility of renal function recovery for patients who require dialysis at disease onset. To investigate this, 40 kidney biopsy-diagnosed ANCA-GN patients who required dialysis at disease onset were enrolled in our study.

## Materials and methods

### Participants

**Fig. 1:**
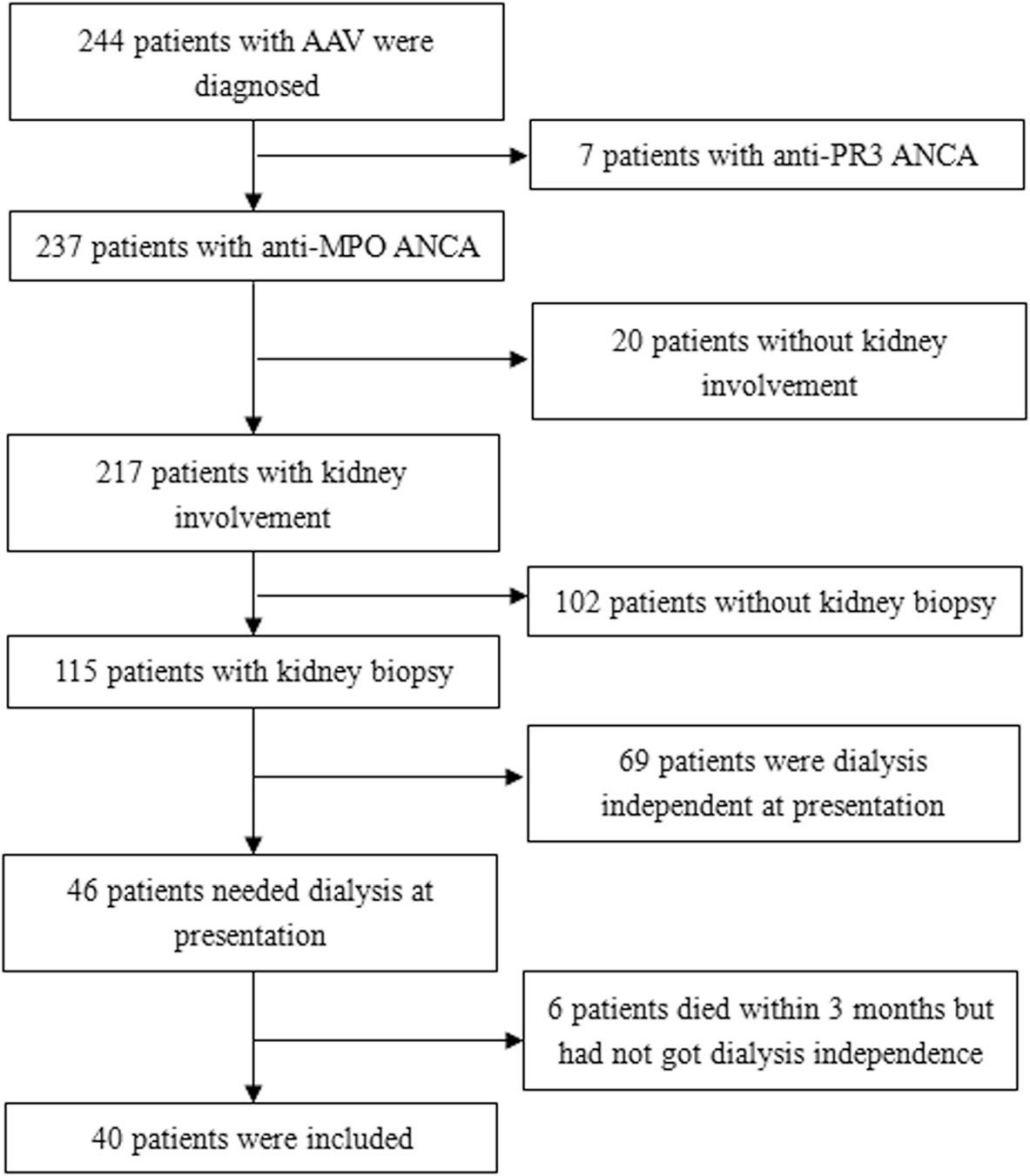
Patients selection flowchart. A total of 244 patients with AAV were diagnosed. Only 7 patients were PR3-ANCA positive and were excluded. Among 237 patients with MPO-ANCA, 217 patients had kidney injury, but 102 patients did not receive kidney biopsy. Patients without kidney biopsy and patients who died before obtaining dialysis independence within 3 months were also excluded. At last, 40 patients were included in this study

Between January 2005 and May 2017, 244 patients with AAV were diagnosed at the Tianjin Medical University General Hospital. All patients fulfilled the Chapel Hill Consensus Conference classification [9]. Only 7 patients were proteinase 3 (PR3)-ANCA positive, all other patients were myeloperoxidase (MPO)-ANCA positive. Patients with PR3-ANCA were excluded because of insufficient numbers. Among the 237 patients with MPO-ANCA, 217 patients had kidney injury (including haematuria, proteinuria and abnormality of kidney function), but 102 patients did not undergo kidney biopsy. Among these 102 patients, 74 patients did not agree to kidney biopsy (Sixteen patients needed dialysis at presentation and 10 patients recovered kidney function within 3 months after treatment) and 28 patients did not receive kidney biopsy because of the insufficient thickness of kidney cortex (Twelve patients needed dialysis at presentation and none recovered kidney function within 3 months following treatment). Among the 115 patients who received kidney biopsy, 46 patients required dialysis at disease onset. In the current study, dialysis dependence was defined as when the patients could not acquire dialysis independence for at least 3 months after treatment, so 6 patients were excluded because they died before getting dialysis independence within 3 months. In summary, 40 patients were included in this study (Fig. 1). The research was in compliance of the declaration of Helsinki and the protocol was approved by the institutional review board of Tianjin Medical University General Hospital. Informed consent was not required because the study was retrospective.

### Clinical and laboratory findings

Clinical data included the following: gender, age (years), time from first symptom (days) and the level of Birmingham Vasculitis Activity Score (BVAS). Blood samples were taken for laboratory test. Estimated glomerular filtration rate (eGFR) was calculated with an equation described previously [10].

### Renal histopathology

Renal biopsies were performed and evaluated using direct immunofluorescence and light microscopy by two pathologists. According to the pathological classification described previously, the biopsy was classified as focal, crescentic, mixed and sclerotic class [8]. The presence of fibrinoid necrosis, cellular crescents and global glomerulosclerosis of each glomerulus was scored. The presence of glomerular lesions was calculated as the percentage of the total number of glomeruli. Interstitial fibrosis (−/+/++), tubular atrophy (−/+/++) and arterial wall thickening (−/+) were scored semiquantitatively [11].

### Treatment

All patients received hemodialysis following admission. Indications of dialysis included serum creatinine > 500 μmol/L, severe electrolyte and acid-base abnormalities, volume overload, pericarditis and encephalopathy [12]. Dialysis was terminated when eGFR surpassed 15 ml/min/1.73m^2^ and there was no hyperkalemia, heart failure or edema. In the induction of remission, all patients received corticosteroid treatment. Oral or intravenous corticosteroid was prescribed at an initial dosage of 1 mg/kg/day for 1 month, with reducing doses over time. Due to the severe kidney injury, it was recommended that all patients receive PE treatment, but due to restrictions in the plasma supply, only 14 patients received PE treatment (2000 ml per treatment session, 3–10 treatment sessions). The other 26 patients who could not be provided enough plasma did not receive PE treatment; they received corticosteroid pulse therapy. Intravenous methylprednisolone pulse therapy (MP) was prescribed for 3 consecutive days (200–500 mg/d) and the MP regimen was repeated after a week if necessary. The majority of patients (31 of 40) received intravenous or oral cyclophosphamide (CTX). 400 mg CTX was administered intravenously every two weeks (Pulse CTX) or by daily oral dose of 50–100 mg according to body weight (Oral CTX). Oral corticosteroid+CTX or corticosteroid+mycophenolate (0.5-2 g/d) was administered for more than 1 year for maintenance therapy. Oral prednisone was prescribed at an initial dosage of 1 mg/kg/day for 1 month, with reducing doses over time. No patient received daily oral azathioprine (AZA) treatment because AZA was not available in our hospital.

### Statistical analyses

Differences of quantitative parameters were assessed using one-way ANOVA tests (for data that were normally distributed) or nonparametric test (for data that were not normally distributed). Categorical variables are presented as frequencies. Survival was calculated with the Kaplan-Meier, and the curves were compared by Log-Rank test. To calculate the area under the curve (AUC) value, receiver operating characteristics (ROC) curves were generated. The cut-off value was confirmed by calculating Youden index. For correlation analysis the spearman test was used. *P* values lower than 0.05 were considered significant. The software SPSS, version 19.0 for Windows (IBM, Chicago, IL, USA), was used for statistical analyses.

## Results

### No differences in baseline clinical test characteristics, with the exception of platelet counts, were observed among patients who acquired complete renal recovery, partial renal recovery, or who remained dialysis dependent

In our study, all patients were dialysis dependent on admission. After standardized treatment, 25 patients obtained dialysis independence within 3 months. Among the 25 patients who got dialysis independence, 13 patients obtained complete renal recovery (CR, eGFR> = 60 mL/min/1.73m^2^) and the other 12 patients acquired partial renal recovery (PR, 15 mL/min/1.73m^2^ < eGFR< 60 mL/min/1.73m^2^). As shown in Table 1, there were no differences of the clinical laboratory characteristics, with the exception of the platelet counts, among the 3 groups. Patients with renal recovery had higher platelet counts (CR: 326.3 ± 160.4 10^9^/L, PR: 269.3 ± 137.1 10^9^/L) than patients who remained dialysis dependent (201.2 ± 66.4 10^9^/L, *P* = 0.040). There was a trend of higher hemoglobin in patients with renal recovery (CR: 8.5 ± 1.7 g/L, PR: 7.9 ± 1.0 g/L) compared to those who remained dialysis dependent (7.3 ± 1.5 g/L), but the difference was not statistically significant (*P* = 0.095).

**Table 1:** Comparison of baseline clinical laboratory characteristics of patients with renal recovery and patients remaining on dialysis within 3 months

Feature	Patients with complete renal recovery (*n* = 13)	Patients with partial renal recovery (*n* = 12)	Patients remaining on dialysis (*n* = 15)	*P* value
Male/female	9/4	6/6	6/9	0.297
Age (years)	63.1 ± 9.5	57.7 ± 7.3	55.9 ± 15.1	0.252
Time from first symptom (days)	70.7 ± 75.9	110.0 ± 112.4	92.1 ± 46.1	0.674
BVAS	23.3 ± 6.3	19.9 ± 5.0	22.0 ± 6.3	0.368
Fever (Y/N)	8/5	8/4	8/7	0.774
Weight loss (Y/N)	5/6	4/8	5/10	0.781
Muscle/joint (Y/N)	3/10	3/9	3/12	0.952
Skin (Y/N)	1/12	0/12	3/12	0.215
Eyes/mucous membranes (Y/N)	3/10	2/10	3/12	0.923
Ear/nose/throat (Y/N)	6/7	5/7	4/11	0.534
Lung (Y/N)	8/5	4/8	7/8	0.368
Cardiovascular (Y/N)	1/12	0/12	0/15	0.345
Digestive tract (Y/N)	2/11	4/8	2/13	0.382
Nervous system (Y/N)	3/10	2/10	2/13	0.792
Gross hematuria (Y/N)	0/13	2/10	1/14	0.283
ANCA level (IU/mL)	89.3 ± 21.7	101.7 ± 36.0	94.9 ± 34.0	0.847
eGFR (mL/min/1.73m^2^)	7.0 ± 2.3	6. 6 ± 2.1	5.7 ± 1.8	0.231
Proteinuria (g/24 h)	2.8 ± 2.3	2.5 ± 3.0	3.5 ± 1.7	0.641
Hemoglobumin (g/L)	8.5 ± 1.7	7.9 ± 1.0	7.3 ± 1.5	0.095
White blood cell (10^9^/L)	9.3 ± 2.7	8.0 ± 3.1	10.2 ± 6.8	0.507
Platelet (10^9^/L)	326.3 ± 160.4	269.3 ± 137.1	201.2 ± 66.4	0.040
ESR (mm/h)	83.2 ± 41.0	71.9 ± 39.4	82.5 ± 45.2	0.756
Serum albumin (g/L)	28.4 ± 5.5	27.3 ± 7.3	28.1 ± 6.9	0.275
Serum calcium (mmol/L)	2.0 ± 0.4	1.9 ± 0.4	1.8 ± 0.4	0.374
Serum phosphate (mmol/L)	1.7 ± 0.4	1.7 ± 0.4	1.7 ± 0.3	0.215
Serum PTH (pg/mL)	128.4 ± 95.3	131.4 ± 86.3	137.0 ± 93.4	0.114
Increased RF (Y/N)	7/6	3/9	3/12	0.130
C-reactive protein (mg/dL)	4.6 (0.9, 15.6)	1.8 (0.2, 8.7)	1.9 (0.3, 16.3)	0.105
Complement 3 (mg/dL)	88.7 ± 33.3	82.7 ± 23.9	89.0 ± 23.6	0.896
Complement 4 (mg/dL)	19.0 ± 8.3	23.8 ± 3.2	30.7 ± 8.0	0.113

*ANCA* Antineutrophil cytoplasmic antibody, *BVAS* Birmingham vasculitis activity score, *eGFR* Estimated glomerular filtration rate, *ESR* Erythrocyte sedimentation rate, *PTH* Parathyroid hormone, *RF* Rheumatoid factor

### Renal histopathological characteristics were strongly associated with the kidney prognosis

All patients received kidney biopsy, and all biopsy specimens contained more than 10 glomeruli. As shown in Table 2, the percentages of normal glomeruli and cellular crescent of patients with CR or PR were significantly higher than the patients who remained dialysis dependent (*P* = 0.007 and *P* = 0.015), while the percentages of fibrous crescent and global glomerulosclerosis of patients with CR or PR were significantly lower than those of patients who remained dialysis dependent (both *P* < 0.001). All patients with PR or patients who remained dialysis dependent had arterial wall thickening, while 38.5% of patients with CR did not have arterial wall thickening (*P* = 0.003).

**Table 2:** Comparison of renal histopathological characteristics of patients with renal recovery and patients remaining on dialysis within 3 months

Feature	Patients with complete renal recovery (n = 13)	Patients with partial renal recovery (*n* = 12)	Patients remaining on dialysis (*n* = 15)	*P* value
Normal glomeruli (%)	24.2 (0.0, 57.2)	11.3 (0.0, 40.0)	0.0 (0.0, 15.2)	0.007
Fibrinoid necrosis (%)	3.8 (0.0, 16.7)	0.0 (0.0, 10.0)	0.0 (0.0, 3.1)	0.065
Cellular crescent (%)	58.1 (19.0, 100.0)	50.8 (20.0, 80.0)	31.7 (3.9, 65.6)	0.015
Fibrous crescent (%)	6.3 (0.0, 40.7)	18.8 (9.7, 31.4)	40.3 (0.0, 82.9)	< 0.001
Global glomerulosclerosis (%)	0.0 (0.0, 33.3)	2.5 (0.0, 14.8)	20.0 (0.0, 40.0)	< 0.001
Interstitial infiltrates (−/+/++/+++)	1/2/6/4	1/0/5/6	0/2/8/5	0.694
Interstitial fibrosis (−/+/++)	5/7/1	2/5/5	1/8/6	0.133
Tubular atrophy (−/+/++)	1/7/5	0/3/9	0/4/11	0.213
Arterial wall thickening (−/+)	5/8	0/12	0/15	0.003

### Mixed class of pathological classification is associated with poor kidney outcome

**Fig. 2:**
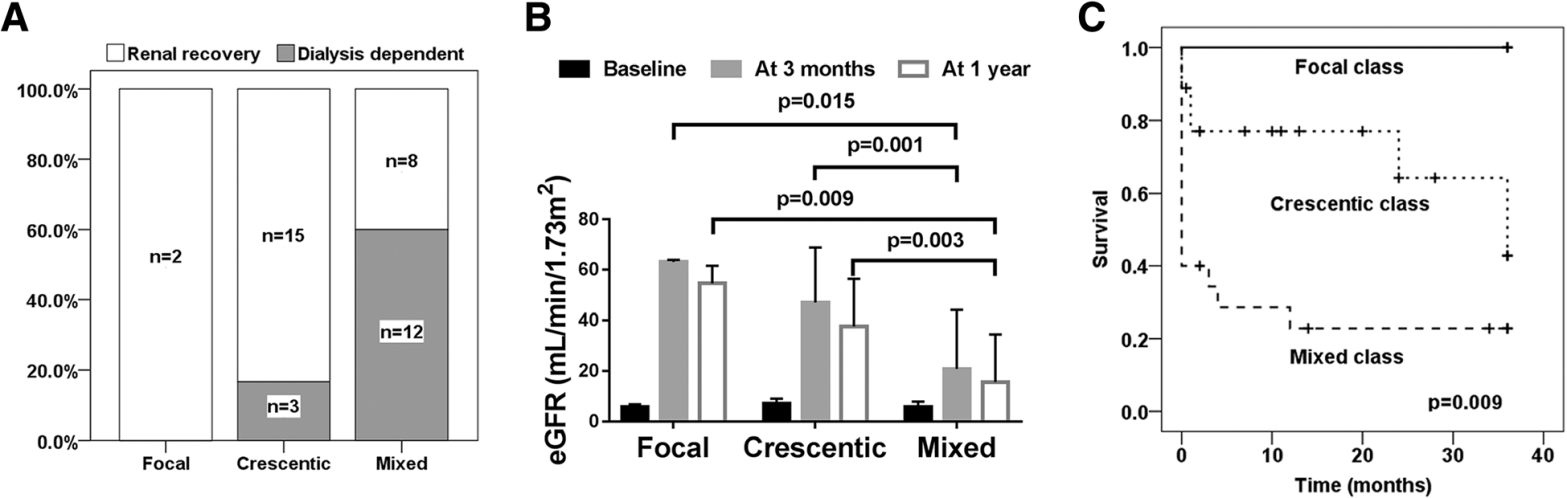
Kidney prognosis of patients with different histopathologic classification. **a** The renal recovery rate of patients with focal, crescentic or mixed class. **b** The estimated glomerular filtration rate (eGFR) at admission, 3 months after therapy and 1 year after therapy of patients with focal, crescentic or mixed class. **c** Comparison of the combined end point (Death and ESRD) among patients with different histopathologic classification. The longest follow-up time was 36 months

The distributions of 4 pathological categories were investigated. No sclerotic class was found among the 40 patients. Only 2 patients were diagnosed as focal class, and both obtained renal recovery within 3 months. Among 18 patients in the crescentic class, 15 acquired renal recovery within 3 months and the other 3 stayed dialysis dependent. Among the 20 patients in the mixed class, 8 got renal recovery and the other 12 kept dialysis dependent. The renal recovery rate of the mixed class was significantly lower than that of crescentic class (*p* = 0.006) (Fig. 2a).

We compared the eGFR at admission, at 3 months, and at 1 year among patients in the different pathological categories. There was no difference in baseline eGFR among patients with focal, crescentic or mixed class. After 3 months of treatment, the eGFR of all 3 groups increased, but the eGFR of patients in the mixed class was lower than that of patients in the crescentic or focal classes. After following up for 1 year, 4 out of 18 patients in the crescentic class died, while 3 out of 20 patients in the mixed class died. Among the living patients, the eGFR of mixed class patients was still lower than that of the crescentic and focal classes (Fig. 2b).

Since 12 patients who recovered renal function obtained PR but not CR, the renal survival of these patients at 1 year was analyzed. We observed that 3 patients with mixed class entered ESRD, and these 3 patients had higher a proportion of global glomerulosclerosis than patients who did not enter ESRD (Table 3).

**Table 3:** Comparison of the baseline renal histopathological characteristics between patients who entered ESRD and who did not enter ESRD after follow-up for 1 year among 12 patients with partial renal recovery

Feature	Patients who entered ESRD (*n* = 3)	Patients who did not enter ESRD (*n* = 9)	*P* value
Normal glomeruli (%)	0.0 (0.0, 4.0)	14.3 (5.0, 36.7)	0.405
Fibrinoid necrosis (%)	0.0 (0.0, 0.0)	0.0 (0.0, 10.0)	0.275
Cellular crescent (%)	48.6 (20.0, 80.0)	51.5 (33.3, 74.2)	0.782
Fibrous crescent (%)	30.0 (13.3, 31.4)	18.2 (9.7, 30.0)	0.195
Global glomerulosclerosis (%)	0.0 (0.0, 14.8)	10.0 (6.7, 14.3)	0.038
Pathological classification (F/C/M/S)	0/0/3/0	0/6/3/0	0.046

*F/C/M/S* Focal/crescentic/mixed/sclerotic

We then compared the combined end point (Death and ESRD) among patients with different pathological categories. As shown in Fig. 2c, the order of the survival was focal, crescentic and mixed classes (*p* = 0.009).

### Percentage of fibrous crescent+global glomerulosclerosis was a marker to predict the kidney outcome

**Fig. 3:**
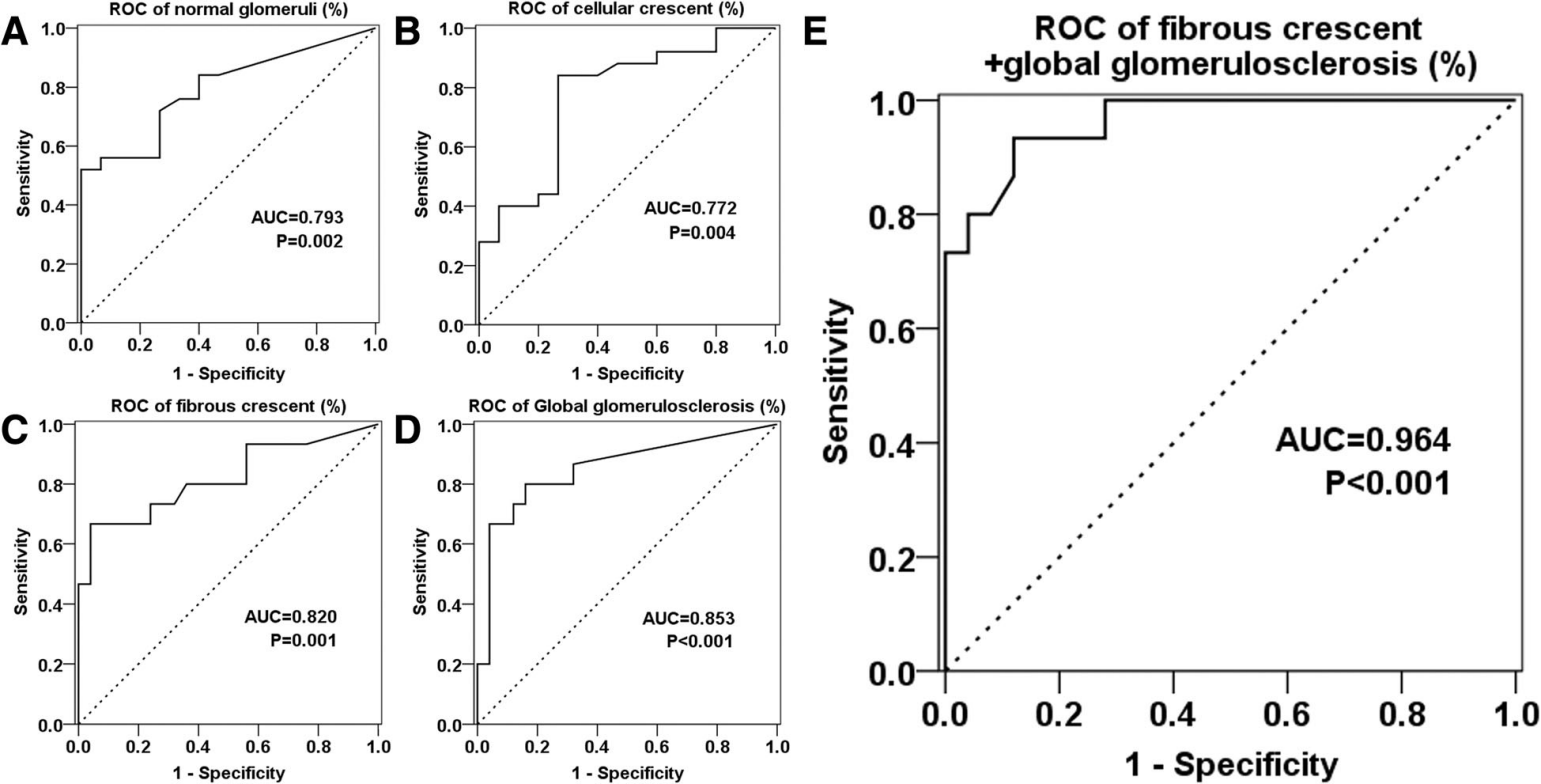
Receiver operating characteristic (ROC) curves of different histopathologic indexes including the percentages of: normal glomeruli (**a**), cellular crescent (**b**), fibrous crescent (**c**), global glomerulosclerosis (**d**) and fibrous crescent+global glomerulosclerosis (**e**). Area under the curve (AUC) was calculated to distinguish patients with renal recovery from patients remaining on dialysis

ROC curves were calculated to explore whether there were pathological parameters which could predict the renal prognosis. The ROC curves are shown in Fig. 3a. The AUC of global glomerulosclerosis was the greatest (0.853) among all 4 glomerular parameters. Since fibrous crescent usually indicates a kind of irreversible lesion, we attempted to determine whether the combination of fibrous crescent and global glomerulosclerosis could increase the accuracy of the diagnosis. As shown in Fig. 3b, the AUC of fibrous crescent+global glomerulosclerosis was greater than any other parameter. When the percentage of fibrous crescent+global glomerulosclerosis was 32.6%, the Youden index was at the peak value (sensitivity was 93.3% and specificity was 88.0%). When the percentage of fibrous crescent+global glomerulosclerosis exceeded 47.9%, renal recovery was unlikely.

Increased peripheral platelet counts were associated with reversible pathological lesions and high renal recovery rate.

**Fig. 4:**
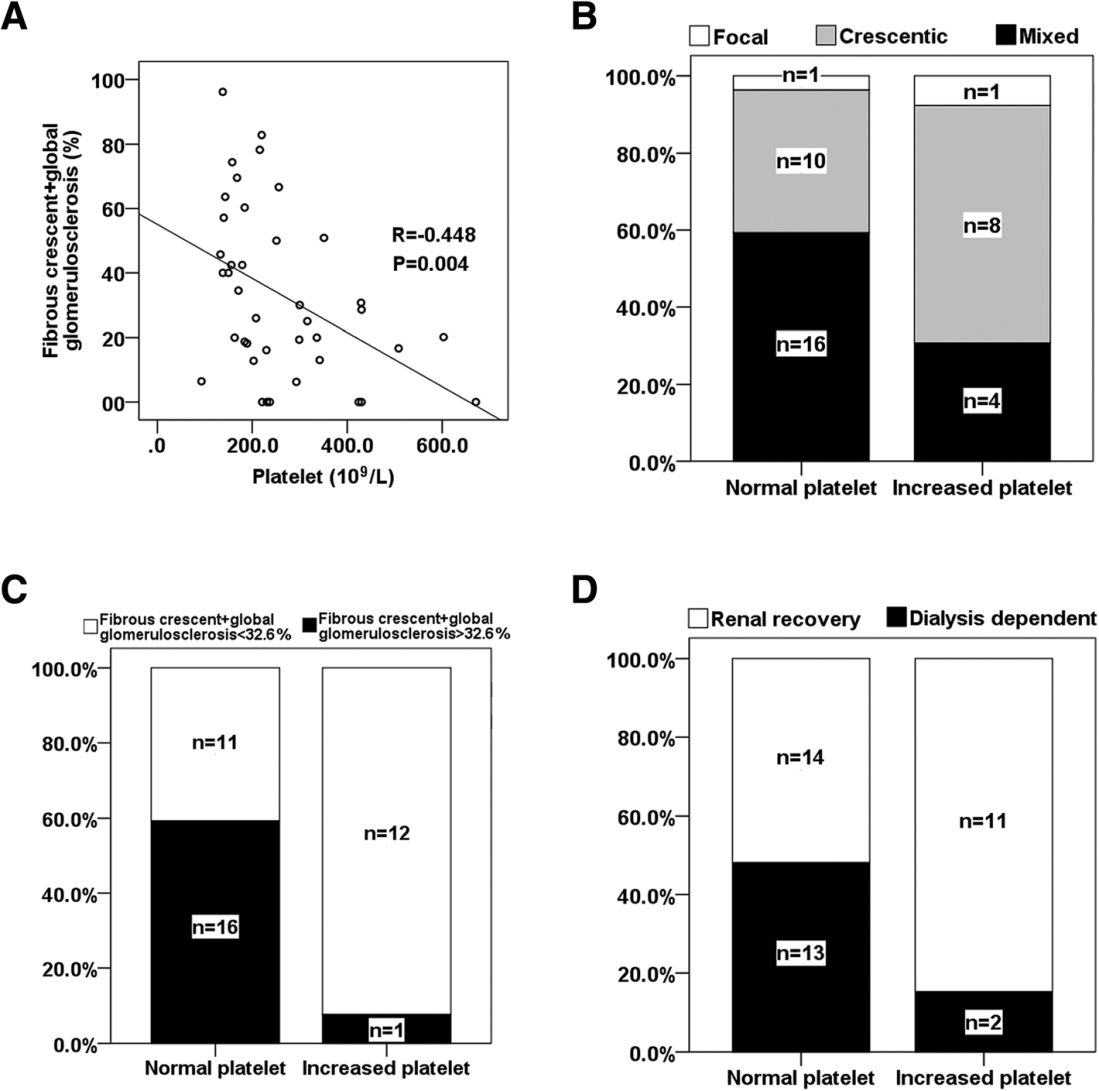
Relationship between platelet counts and histopathologic indexes and relationship between platelet counts and prognosis. **a** Correlation between platelet counts and the percentages of fibrous crescent+global glomerulosclerosis. **b** Comparison of the difference of histopathologic classification between patients with normal and increased platelets. Increased platelets were diagnosed when platelets were more than the upper limit 300 × 10^9^/L. **c** Comparison of the proportions of patients with fibrous crescent+global glomerulosclerosis more than cut-off value (32.6%) between patients with normal and increased platelets. **d** Comparison of the proportions of renal recovery within 3 months between patients with normal and increased platelets

Since patients who obtained renal recovery had higher platelet counts than patients who were dialysis dependent, we explored whether platelet counts could be used as a substitute for pathological lesions to determine kidney outcome. Correlation analysis indicated that platelet counts negatively correlated with the percentage of fibrous crescent+global glomerulosclerosis (Fig. 4a). There were no differences in the distributions of focal, crescentic and mixed categories between patients with normal platelets and patients with increased platelets (Increased platelets meant the platelet count was larger than the upper limit of normal range 300 × 10^9^/L) (Fig. 4b, *p* = 0.238), but for the majority patients with increased platelets, the percentages of fibrous crescent+global glomerulosclerosis were less than 32.6% (Fig. 4c, *p* = 0.002, compared with patients with normal platelets). Correspondingly, most patients with increased platelets obtained renal recovery (CR + PR) within 3 months (Fig. 4d, *p* = 0.045, compared with patients with normal platelets). However, the AUC of platelet counts for distinguishing renal recovery from dialysis dependence was only 0.709.

### PE treatment accelerated the renal recovery

Effects of different therapy regimens on renal recovery were analyzed. All 40 patients received daily oral corticosteroid as a primary treatment. Among these patients, 7 received MP + pulse CTX treatment, 19 patients received MP + oral CTX treatment, 3 patients received PE + pulse CTX treatment and 11 patients received PE + oral CTX treatment. As shown in Table 4, there were no significant differences in the baseline BVAS, eGFR, proteinuria, or incidence of gross hematuria between the MP and PE groups or between pulse CTX and oral CTX groups. There were also no significant differences in kidney histopathology parameters among groups (data not shown). Compared with MP, PE accelerated the rate of renal recovery (29.4 ± 15.6 vs. 41.4 ± 11.7 days, *p* = 0.039), and there was a tendency for a greater renal recovery rate in PE group (79%) compared with MP group (53%).

**Table 4:** Influences of different therapy regimens on the renal recovery within 3 months

	MP vs. PE			Pulse CTX vs. Oral CTX		
	MP (*n* = 26)	PE (*n* = 14)	*P* value	Pulse CTX (*n* = 10)	Oral CTX (*n* = 30)	*P* value
MP/PE	–	–	–	7/3	19/11	0.702
Pulse CTX/Oral CTX	7/19	3/11	0.702	–	–	–
BVAS	22.9 ± 6.2	19.9 ± 5.1	0.132	23.4 ± 4.9	21.3 ± 6.2	0.333
eGFR (mL/min/1.73 m2)	6.4 ± 2.0	6.2 ± 2.3	0.774	6.6 ± 2.1	6.3 ± 2.1	0.646
Proteinuria (g/24 h)	2.7 ± 1.7	3.3 ± 3.2	0.384	2.8 ± 2.1	3.0 ± 2.5	0.848
Gross hematuria (Y/N)	3/23	0/14	0.186	1/9	2/28	0.729
Renal recovery (CR/PR/N)	7/7/12	6/5/3	0.297	3/4/3	10/8/12	0.715
Time for getting dialysis independence (days)	41.4 ± 11.7	29.4 ± 15.6	0.039	34.8 ± 11.1	36.3 ± 15.9	0.894

*BVAS* Birmingham vasculitis activity score, *CTX* Cyclophosphamide, *CR* Complete remission, *eGFR* Estimated glomerular filtration rate, *MP* Methylprednisolone pulse therapy, *PE* Plasma exchange, *PR* Partial remission

## Discussion

RPGN is the clinical manifestation of several types of kidney diseases, including anti-glomerular basement membrane antibody disease (type 1 RPGN), AAV (type 2 RPGN), and RPGN with glomerular deposition of immune complexes (type 3 RPGN) [13]. Patients requiring dialysis at disease onset caused by type 1 RPGN usually rapidly enter ESRD [14, 15]. In contrast with type 1 RPGN, most patients with type 3 RPGN usually develop ESRD gradually. As for type 2 RPGN (ANCA-GN), the kidney outcome varies for different individuals.

In the current study, we found that pathological severity was the most important factor affecting renal outcome. Currently, the only practical tool for estimating the renal prognosis of ANCA-GN is the histopathologic classification proposed in 2010 [8]. Its clinical value has been evaluated by several studies in recent years [16–29]. Interestingly, the distribution of the four renal histological categories in our study is different from all previous studies. In the previous studies, the 4 pathological categories were approximately evenly spread, while in our cohort the crescent and mixed classes accounted for a near majority of patients. Although the initial study of Berden AE et al. suggested the prognosis of the mixed class was worse than that of the crescentic class [8], there is no consistent conclusion in subsequent validation studies concerning the comparison of the prognosis between the mixed and crescentic classes. In our study, the renal outcome of the mixed class was significantly worse than that of the crescentic class.

According to previous studies, the sclerotic class (glomerulosclerosis over 50%) had the worst prognosis in ANCA-GN, but most patients took several years to develop ESRD. However, the patients in our study developed ESRD directly when fibrous crescent+glomerulosclerosis was greater than 32.6%. These results were very interesting and required interpretation. Theoretically, not all glomeruli with cellular crescents could return to normal even following intensive immunosuppressive treatments. There were only two patients in the focal class in this study, and we speculate that the proportions of normal glomeruli in both the mixed and crescentic classes in our study were much lower than the patients of the mixed and crescentic classes in previous studies. In contrast, since the proportion of chronic lesions was greater in the mixed class than in the crescentic class, it was not difficult to see why the renal prognosis of the mixed class was inferior to that of the crescentic class in our study. Therefore, for patients needing dialysis at disease onset, the renal outcomes of the crescentic and mixed classes are significantly different.

In clinical work, not all patients are able to undergo kidney biopsy, so an alternative biomarker is of great importance [30, 31]. Platelet count has not been a significant factor in previous studies, but these studies included both patients needing dialysis and patients who did not need dialysis. In our study, platelet count was the only laboratory parameter which exhibited a significant difference between patients who obtained renal recovery and those who remained dialysis dependent. It is noteworthy that platelet counts have been associated with disease activity in AAV and can distinguish acute infection from active disease [32, 33]. Platelet-leukocyte aggregates were significantly higher thrombocytosis. Most importantly, platelets could activate the alternative complement pathway, which is crucial in the pathogenesis of AAV [34]. The peripheral levels of platelet-derived microparticles (PMPs) were significantly associated with disease activity and the proportion of crescents in the renal specimen [35]. We found platelet counts correlated with the percentages of fibrous crescent+global glomerulosclerosis. However, there was not a suitable cut-off value for platelet counts to predict kidney outcome. In addition, the diagnosis of thrombocytosis was influenced by the definition of upper normal limit of platelets. In our hospital, thrombocytosis was diagnosed when platelet counts were greater than 300 × 10^9^/L. Thus, platelet counts cannot replace pathological parameters to predict renal prognosis in AAV.

CRP is a classical acute-phase protein. Interestingly, although there was a correlation between platelets and CRP (data not shown), there was no significant difference in CRP among patients with different renal outcomes. One possible explanation is that CRP is more easily affected by other factors (such as potential infection) than platelets [32].

In the treatment of ANCA-GN, PE has been recommended due to its superiority to MP [7]. In our study, we also demonstrated that patients receiving PE had a faster renal recovery than patients receiving MP, but a higher remission rate was not observed. This may be due to the small cohort size of our study. However, the largest trial of AAV to date (PEXIVAS study), which included 704 participants from 98 sites in 15 countries, indicated no improvement in outcomes (death or ESRD) of patients who received PE. This issue requires further investigation.

Several limitations of our study should be mentioned. Firstly, due to the restrictions of the enrollment standard, the sample size of the study was small. In our study, no sclerotic class patients were identified, but as many as twenty-eight patients who were dialysis dependent at disease onset were excluded because they did not receive kidney biopsy. Among these 28 patients, 12 patients had kidney atrophy and did not recover kidney function within 3 months. We could not exclude the possibility that most, or even all of these patients were of the sclerotic class. Secondly, only MPO-ANCA positive patients were included because PR3-ANCA positive AAV is relatively rare in our center. So whether the present results pertain to patients with PR3-ANCA positive AAV is unclear. Thirdly, being limited by the inclusion standard, all patients without kidney biopsy or patients that died within 3 months were excluded. This affected the estimation of the effects of different therapies on renal recovery. Finally, whether patients received either MP or PE treatment was not randomly determined due to the shortage of plasma.

## Conclusions

In conclusion, for ANCA-GN requiring dialysis at disease onset, the crescentic and mixed classes account for the majority of patients in our cohort. Mixed class is associated with worse prognosis than crescentic class. A high level of fibrous crescent+global glomerulosclerosis pathology is a predictor of dialysis dependence. Patients with increased platelet counts have a greater number of acute pathological lesions and higher renal recovery rate compared to patients with normal platelet counts.

## Data Availability

The datasets used and/or analyzed during the current study are available from the corresponding author on reasonable request.

## Abbreviations

AAV: Anti-neutrophil cytoplasmic antibody-associated vasculitis
ANCA: Anti-neutrophil cytoplasmic antibody
AUC: Area under the curve
BVAS: Birmingham Vasculitis Activity Score
CR: Complete renal recovery
CTX: Cyclophosphamide
eGFR: Estimated glomerular filtration rate
ESRD: End stage renal disease
GN: Glomerulonephritis
MP: Methylprednisolone pulse therapy
MPO: Myeloperoxidase
PE: Plasma exchange
PMPs: platelet-derived microparticles
PR: Partial renal recovery
PR3: Proteinase 3
ROC: Receiver operating characteristics
RPGN: Rapidly progressive glomerulonephritis
